# The impact of workload and emotional demands on turnover intentions: the mediating and moderating effects of job burnout

**DOI:** 10.3389/fpsyg.2025.1699421

**Published:** 2025-12-01

**Authors:** Wen-Yu Hung, I-Hsiung Chang, Yueh-Chih Hsiao

**Affiliations:** 1 Graduate Institute of Technological and Vocational Education, National Taipei University of Technology, Taipei, Taiwan; 2 Department of Child Care and Industries, Fooyin University, Kaohsiung, Taiwan; 3 Department of Technology Application and Human Resource Development, College of Technology and Engineering, National Taiwan Normal University, Taipei, Taiwan

**Keywords:** workload, emotional demands, job burnout, turnover intention, Job Demands-Resources theory, Conservation of Resources theory

## Abstract

**Introduction:**

Although workload and emotional demands are recognized as key antecedents of job burnout, research focusing on preschool teachers has rarely compared their relative effects or explored whether burnout may function simultaneously as a mediator and a moderator. Using the Job Demands-Resources (JD-R) and Conservation of Resources (COR) frameworks, this study examines how these job demands influence preschool teachers' turnover intention through job burnout, and whether burnout amplifies these associations.

**Methods:**

A two-stage survey was administered to 200 preschool teachers in Taiwan. Measures included workload, emotional demands, job burnout, and turnover intention. Data were analyzed using mediation and moderation analysis.

**Results:**

Workload and emotional demands both significantly and positively predicted turnover intention through job burnout. Moreover, job burnout moderated the effect of workload on turnover intention, with the relationship becoming stronger at higher burnout levels.

**Discussion:**

These findings underscore the dual role of job burnout and highlight the need for early childhood education administrators to reduce job demands, implement burnout prevention strategies, and stabilize the preschool teaching workforce.

## 1 Introduction

In recent years, Taiwan has been facing a declining birthrate, which has led parents to place increasing emphasis on the care and education of each individual child. As a result, expectations regarding the quality of early childhood education and care services have risen significantly. Preschool teachers are not only responsible for daily teaching and caregiving tasks, but also expected to meet parents' high demands concerning learning outcomes, instructional approaches, parent–teacher communication, and timely responses (Chang et al., 2025; Yang et al., 2018). These increasing expectations have intensified both the emotional labor and overall work stress experienced by preschool educators. In addition, preschool teachers in Taiwan commonly face structural challenges such as low salaries, long working hours, insufficient teaching resources, and limited opportunities for career advancement (Hsu et al., 2025; Yeh and Lo, 2024). These issues contribute to generally low job satisfaction and a growing tendency toward turnover.

Turnover intention refers to employees' tendency and willingness to leave their job or profession (Tett and Meyer, 1993). Existing research has confirmed that high turnover rates among teachers not only increase operational costs for organizations (Bassok et al., 2021) and reduce the quality of education (McCormick et al., 2022) but also negatively affect children's development (Schaack et al., 2020). Therefore, understanding the antecedents and mechanisms of turnover intention among preschool teachers and taking measures to reduce turnover intentions has become a critical issue for education managers.

Previous studies have demonstrated that job burnout is a critical factor influencing preschool teachers‘ turnover intention (Li and Yao, 2022; Madigan and Kim, 2021). Defined as a psychological syndrome resulting from prolonged occupational stress, job burnout is typically characterized by emotional exhaustion, depersonalization, and a diminished sense of personal accomplishment (Maslach et al., 2001). Given its substantial negative impact on teachers' job satisfaction as well as their mental and physical health, it is important not only to recognize the role of burnout but also to examine its antecedents more closely.

In today's high-pressure work environment, workload and emotional demands are two primary antecedents of burnout, particularly in preschool education (Heffernan et al., 2022). Workload involves tasks such as lesson planning, classroom management, and participation in curriculum initiatives (Cheng et al., 2025; Pan et al., 2022; Schaack et al., 2020), while emotional demands require ongoing patience, empathy, and regulation in response to children's emotional needs (Lee, 2019; Yin, 2015). Although existing research has examined these stressors individually, few studies have explored their combined or comparative effects on job burnout. Emotional demands, in particular, may exert a stronger influence, given the sustained emotional regulation required in early childhood settings (Näring et al., 2006; Yin et al., 2023). To address this gap, this study adopts the health impairment pathway of the Job Demands–Resources (JD-R) theory (Bakker and Demerouti, 2017; Bakker et al., 2007) to examine how workload and emotional demands jointly contribute to burnout. Furthermore, it aims to compare their relative impact on both burnout and turnover intention, offering a more nuanced understanding of how distinct job demands shape preschool teachers' burnout and turnover intention.

While job burnout has been widely recognized as a key mechanism linking job demands to turnover intention, existing research has primarily emphasized its mediating role, often overlooking the possibility that burnout may also moderate this relationship (Chen et al., 2025). However, some recent studies suggest that a psychological state such as burnout may not only arise from stressors but also exacerbate their negative effects, especially when individuals are already operating with depleted psychological resources (Osei et al., 2024; Yang et al., 2021). Building on this perspective, the present study adopts the Conservation of Resources (COR) theory (Hobfoll, 1989; Hobfoll et al., 2018) to propose that job burnout may serve a dual role—functioning both as a mediator and a moderator in the relationship between job demands and turnover intention. This dual-role approach offers a more dynamic understanding of how stress accumulates and manifests in behavioral outcomes, particularly in high-stress professions like preschool education. Addressing this gap, the current research contributes to a more integrated theoretical model and provides insights for more targeted interventions.

## 2 Theoretical background

### 2.1 The relationship between workload, emotional demands, and job burnout

In today's highly competitive work environment, the significant increase in workload has become a common phenomenon across many sectors, including early childhood education. In the childcare profession, both workload and emotional demands are frequent and unavoidable sources of work-related stress (Hsu et al., 2025; Yeh and Lo, 2024). If these pressures are not properly managed, they can lead to chronic strain and eventually result in job burnout.

Workload typically refers to the number, scope, and complexity of tasks employees are expected to complete within a given timeframe, and it may include physical, cognitive, and emotional components (Bakker et al., 2005). Excessive workload is strongly associated with feelings of being overwhelmed, fatigue, and difficulty maintaining sustained performance (Cheng et al., 2025; Fernet et al., 2004; Schaufeli et al., 2009). In the early childhood education setting, this includes not only classroom instruction and child supervision, but also lesson planning, administrative tasks, and participation in curriculum improvement initiatives.

Emotional demands, on the other hand, refer to the psychological effort required to display or suppress emotions in accordance with organizational expectations, particularly in interpersonal interactions (Zapf et al., 2001). For preschool teachers, this includes demonstrating patience and empathy when managing young children's emotional fluctuations, as well as maintaining professionalism in interactions with parents. Sustained emotional labor has been shown to deplete emotional energy, often resulting in emotional exhaustion and diminished well-being (Brotheridge and Grandey, 2002; Lee, 2019).

This study is grounded in the Job Demands–Resources (JD-R) theory, a widely used framework for understanding occupational stress and employee well-being across professions. The JD-R theory categorizes all workplace characteristics into two broad domains: job demands and job resources (Bakker and Demerouti, 2007; Cheng et al., 2023). Job demands are aspects of the job that require sustained physical, cognitive, or emotional effort, and are therefore associated with physiological and psychological costs. In contrast, job resources refer to aspects of the job that help employees achieve work goals, reduce job demands, or stimulate personal growth and development (Bakker and Demerouti, 2017, 2024).

The JD-R theory proposes two main psychological mechanisms: the health impairment process and the motivational process (Bakker et al., 2005, 2007; Schaufeli and Bakker, 2004). This study focuses on the health impairment pathway, which posits that excessive or prolonged exposure to high job demands can lead to energy depletion and job burnout, particularly when resources are insufficient to offset the stressors (Bakker and Demerouti, 2017, 2024). Under this framework, both workload and emotional demands are considered high-strain demands that contribute directly to the development of burnout. Burnout, in turn, can result in lower job satisfaction, diminished performance, and increased turnover intention.

Empirical research has consistently supported these relationships. High workload has been linked to elevated levels of emotional exhaustion and depersonalization (Cheng et al., 2023; Creagh et al., 2023; Heffernan et al., 2022; Van Droogenbroeck et al., 2014), while emotional demands have been shown to drain emotional resources and lead to burnout over time (Aboagye et al., 2021, 2023; Bodenheimer and Shuster, 2020). When both physical and emotional demands exceed an individual's capacity for coping and recovery, job burnout becomes a likely outcome. Therefore, based on the JD-R theory and previous empirical evidence, this study proposes the following hypotheses:

**H1: Workload has a significant positive impact on job burnout**.
**H2: Emotional demands have a significant positive impact on job burnout**.

### 2.2 The mediating effect of job burnout

Empirical studies have provided substantial support for the mediating role of job burnout in the relationship between job demands and turnover intention. Prior meta-analysis and longitudinal studies have shown that excessive workload is strongly associated with increased emotional exhaustion, which in turn leads to higher levels of burnout and subsequently greater turnover intention (Alarcon, 2011; Cheng et al., 2023; Rajendran et al., 2020). This suggests that when employees are repeatedly overloaded with complex or demanding tasks, their psychological resources become depleted, prompting emotional strain that weakens their attachment to the organization and increases their desire to leave.

In a similar vein, emotional demands have also been found to influence turnover intention through burnout. The continuous requirement to display empathy, suppress negative emotions, and maintain emotional composure in stressful interactions can lead to emotional depletion over time. This pattern has been demonstrated in studies indicating that emotional labor contributes to job burnout, which then increases turnover intention (Lee, 2019; Madigan and Kim, 2021; Li and Yao, 2022). These findings are particularly relevant in early childhood education, where both workload and emotional demands are persistent and demanding.

These findings underscore that job burnout serves as a psychological mechanism that transmits the effects of both workload and emotional demands on turnover intention, especially in high-demand professions such as preschool education. Based on these theoretical and empirical insights, the following hypotheses are proposed:

**H3: Workload has a significant positive impact on turnover intention through job burnout**.
**H4: Emotional demands have a significant positive impact on turnover intention through job burnout**.

### 2.3 The moderating effect of job burnout

Previous studies have consistently emphasized the critical role of job burnout in organizational contexts and have generally regarded it as an important mediating mechanism through which job stress influences outcome variables. However, most prior research has primarily focused on the mediating function of burnout while overlooking the possibility that burnout may also exert a moderating influence (Chen et al., 2025). In fact, existing evidence suggests that a single psychological construct may simultaneously serve as both a mediator and a moderator, depending on its position within the stress–strain process. For instance, Pretorius (2020) found that individuals' problem-solving ability not only mediated the relationship between role ambiguity and emotional exhaustion but also moderated the strength of that relationship. Similarly, Mäntymäki et al. (2022) demonstrated that psychological detachment both attenuated the negative effects of work–family conflict on well-being and transmitted its influence through underlying psychological mechanisms. These findings indicate that mediating mechanisms may also exhibit boundary effects, thereby displaying dual functions of mediation and moderation.

Related research has revealed that job burnout is not merely an outcome of job demands but can also exacerbate employees' perceptions of work stress and workload, creating a self-reinforcing vicious cycle (Maslach and Leiter, 2016). In a 6-month, two-wave longitudinal study, Huang et al. (2015) confirmed the reciprocal relationship between workload and job burnout, showing that heavy workloads increase emotional exhaustion, which in turn heightens perceived work stress. Likewise, Jeung et al. (2018) reported that high levels of burnout make emotional labor more difficult, thereby intensifying employees' psychological stress and emotional demands.

Empirical studies have further examined the moderating effects of job burnout. Yang et al. (2021) found that job burnout negatively moderated the relationship between safety compliance and safety participation—specifically, when burnout was high, employees were more prone to exhibit deviant or unsafe behaviors. In the healthcare sector, Osei et al. (2024) also confirmed that job burnout strengthened the negative relationship between job satisfaction and turnover intention, indicating that highly burned-out employees were more likely to develop turnover intentions when their job satisfaction decreased.

From the perspective of the Conservation of Resources (COR) theory, prolonged exposure to high work demands leads to continuous resource depletion, which triggers a series of negative cycles referred to as the “spiral of resource loss” (Hobfoll, 1989; Hobfoll et al., 2018). Within this process, excessive workloads consume employees' psychological and emotional resources, resulting in burnout, which subsequently undermines their coping capacity and self-efficacy. This, in turn, amplifies their perceptions of work stress and emotional demands (Huang et al., 2015; Jeung et al., 2018). In other words, burnout is not only a consequence of resource loss but also an accelerating force that perpetuates this depletion process, making it more difficult for employees to recover from stress and further impairing their physical and mental health as well as their work performance (Maslach and Leiter, 2016).

Taken together, when employees fall into this spiral of resource loss, additional job demands or emotional challenges tend to be magnified, producing a stress amplification effect. This heightened strain not only increases their psychological burden but also strengthens the positive relationship between job stressors and turnover intention. Conversely, employees with lower levels of burnout retain more psychological and emotional resources, enabling them to better regulate stress responses and buffer against turnover intention. Based on the empirical evidence and the theoretical assumptions of COR theory, proposes the following hypotheses:

**H5: Job burnout moderates the relationship between workload and turnover intention. The higher the job burnout, the stronger the relationship between workload and turnover intention; conversely, the lower the job burnout, the weaker the relationship between workload and turnover intention**.
**H6: Job burnout moderates the relationship between emotional demands and turnover intention. The higher the job burnout, the stronger the relationship between emotional demands and turnover intention; conversely, the lower the job burnout, the weaker the relationship between emotional demands and turnover intention**.

**Figure 1:**
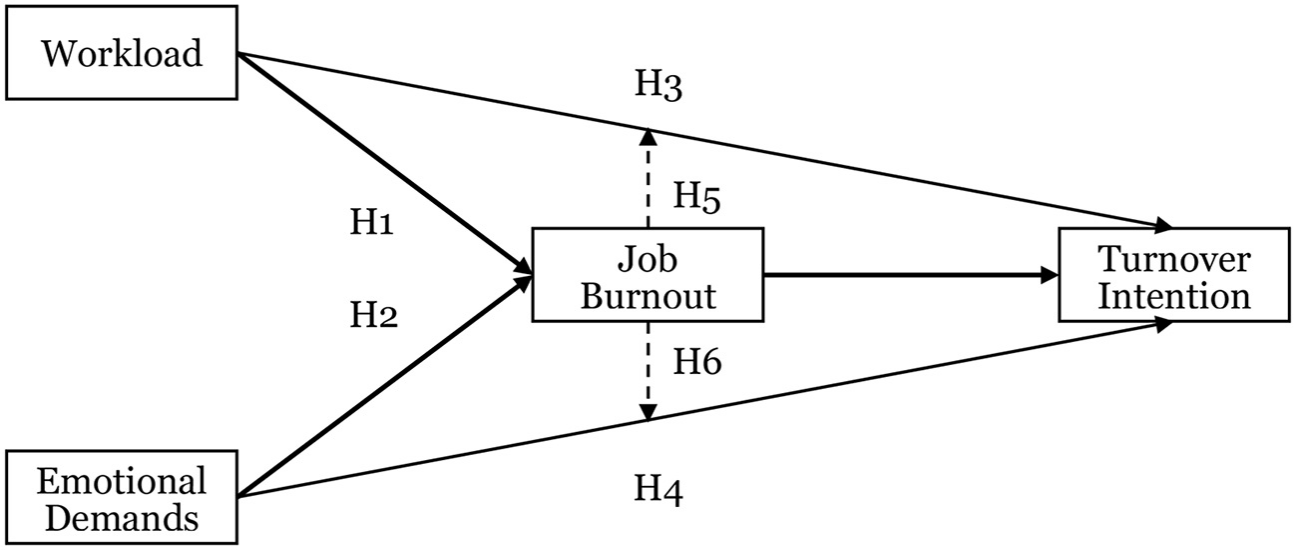
Research framework. Normal solid arrows indicate direct effects; bold solid arrows represent mediating effects through job burnout; dashed arrows indicate moderating effects of job burnout on the relationships between workload, emotional demands, and turnover intention.

In summary, the research framework and hypotheses of this study are illustrated in Figure 1.

## 3 Materials and methods

### 3.1 Research participants and procedure

This study employed a convenience sampling method, targeting childcare workers in Taiwan as the research participants. To ensure that the respondents met the inclusion criteria, questionnaires were distributed through early childhood educators who assisted in forwarding the survey to colleagues within their professional networks. This approach helped confirm that participants were qualified childcare teachers currently working in early childhood institutions, thereby improving the appropriateness of the convenience sample.

To minimize common method variance (CMV), data were collected in two stages using an online survey. Participants provided their email addresses so that their responses could be matched between the two stages. The survey design ensured anonymity, and no personal identifiers were collected to reduce potential response bias. Additionally, the purpose of the study and the variable names were not displayed in the questionnaire, following the item meaning obscuration procedure proposed by Podsakoff et al. (2003), which helps mitigate the effects of CMV.

During the Time 1 data collection stage, participants completed items measuring workload and emotional demands, resulting in 288 responses. Three weeks later, the Time 2 stage was conducted to assess job burnout and turnover intention, yielding 230 responses. After removing incomplete and inconsistent questionnaires, a total of 200 valid responses were retained for statistical analysis.

The final sample size met the recommended thresholds for structural equation modeling (SEM). According to Hair et al. (2006), SEM generally requires a minimum of 150 to 200 cases to ensure stable parameter estimates. In addition, the sample size should be five to ten times the total number of measurement items. Given that the current study included 21 measurement items, an appropriate sample size would range from 105 to 210 participants. Therefore, the final sample of 200 respondents was deemed adequate for the analysis conducted in this study.

Regarding the sample characteristics, the largest age group was 41–50 years old, accounting for 31.5% of the total participants. In terms of education, most respondents held a bachelor's degree in early childhood education (62.5%). With respect to marital status, 62.5% of the participants were married. As for Job tenure, the largest proportion (37.5%) had between five and ten years of work experience. Lastly, nearly half of the respondents (47.0%) held the position of childcare teacher (see Table 1).

**Table 1:** Demographic descriptive statistics.

**Variable**	**Category**	***N***	**%**
Age	Under 30	60	30
	31–40	26	13
	41–50	63	31.5
	51 and above	51	25.5
Education	Junior college	14	7
	Bachelor's degree (Non–early childhood education major)	18	9
	Bachelor's degree (Early childhood education major)	125	62.5
	Master's degree	43	21.5
Marital status	Single	75	37.5
	Married	125	62.5
Job tenure	Less than 2 years	54	27
	2–5 years	25	12.5
	5–10 years	75	37.5
	10–20 years	36	18
	More than 20 years	10	5
Position	Substitute childcare teacher	7	3.5
	Assistant childcare teacher	14	7
	Childcare teacher	94	47
	Childcare teacher and director	58	29
	Principal	27	13.5

*N* = 200.

Percentages are based on valid responses.

### 3.2 Measures

This study utilized a seven-point Likert scale for the questionnaire responses, ranging from strongly disagree (1) to strongly agree (7), with higher scores indicating greater agreement with the corresponding statements. The definitions and operationalizations of the variables are as follows:

Workload scale was adapted from Shantz et al. (2016) and consists of 6 items measuring the workload of childcare workers. Example items include: “I always feel that I don't have enough time to complete all my tasks” and “I often feel that I have too much work to handle alone.” The overall Cronbach's α for the scale was 0.916.

Emotional demands scale was adapted from Yin (2015) and consists of 4 items measuring the duration and intensity of emotional expression and regulation required in work-related interactions for childcare workers. Example items include: “To do my job well, I need to spend time interacting with others (such as students, parents, and colleagues)” and “To reassure students and parents, I need to manage my emotions and behavior.” The overall Cronbach's α for the scale was 0.801.

Job burnout scale was adapted from Maslach and Jackson (1981) and consists of 9 items measuring job burnout among childcare workers. Example items include: “I feel frustrated with my job” and “I feel emotionally drained from my work.” The overall Cronbach's α for the scale was 0.909.

Turnover intention scale was adapted from Shore et al. (1990) and consists of 3 items measuring the extent to which childcare workers are considering leaving their current job. Example items include: “If possible, I would like to change jobs,” “I often think about quitting,” and “I might try to change my workplace within the next year.” The overall Cronbach's α for the scale was 0.717.

To rule out potential confounding factors, this study included control variables based on previous research. Studies have shown that older employees typically have lower turnover intentions (Ng and Feldman, 2010). Additionally, employees with longer tenure tend to be more familiar with and adapted to their current work environment, leading to lower turnover intentions (Griffeth et al., 2000). Finally, employees in higher positions tend to exhibit stronger organizational commitment and receive better benefits, which reduces turnover intentions (Lambert et al., 2001). Therefore, age, job tenure, and position were controlled in this study.

### 3.3 Common method variance

Despite the steps taken to minimize common method variance, such as using two-wave data collection, respondent anonymity techniques, and item meaning obscuration, it cannot be entirely eliminated. Thus, following the recommendations of Podsakoff et al. (2003), *post-hoc* testing was conducted. The Harman single-factor test was used, with all items related to workload, emotional demands, job burnout, and turnover intention included in the analysis. The total variance explained by a single factor without rotation was 32.60%, indicating that common method variance was not a serious issue in this study.

### 3.4 Data analysis

Following the recommendations of Anderson and Gerbing (1988), a two-step approach was employed for Structural Equation Modeling (SEM). This study used JASP 17.3 to conduct Confirmatory Factor Analysis (CFA) to examine the convergent and discriminant validity of the research variables. The model fit indices of the theoretical model were compared with those of alternative competing models. Additionally, hierarchical regression analysis was conducted using SPSS 25.0 to test the research hypotheses.

## 4 Results

### 4.1 Confirmatory factor analysis

This study examined the convergent validity of the research model using CFA. As presented in Table 2, the factor loadings for each research variable ranged from 0.658 to 0.850, exceeding the recommended threshold of 0.5 (Hair et al., 2006), indicating good representativeness of the items. Although the average variance extracted (AVE) for “turnover intention” did not reach the ideal value of 0.5, its value was 0.476, which is close to the standard. According to Fornell and Larcker (1981), when AVE is slightly below 0.5, but the composite reliability (CR) exceeds 0.6, the variable can still be considered to have convergent validity. In this study, all CR values for the research variables exceeded 0.6, confirming that the model exhibits good convergent validity.

**Table 2:** Measurement model.

**Factors**	**Items**	**Factor loading**	**SMC**	**CR**	**AVE**
Workload	Loading 1 (R)	0.741	0.549	0.917	0.648
	Loading 2	0.777	0.604		
	Loading 3	0.820	0.672		
	Loading 4	0.850	0.723		
	Loading 5	0.791	0.626		
	Loading 6	0.839	0.704		
Emotional demands	Emd 1	0.691	0.477	0.806	0.510
	Emd 2	0.776	0.602		
	Emd 3	0.684	0.468		
	Emd 4	0.699	0.489		
Job burnout	Burnout 1	0.739	0.546	0.910	0.528
	Burnout 2	0.697	0.486		
	Burnout 3	0.786	0.618		
	Burnout 4	0.722	0.521		
	Burnout 5	0.771	0.594		
	Burnout 6	0.753	0.567		
	Burnout 7	0.665	0.442		
	Burnout 8	0.695	0.483		
	Burnout 9	0.713	0.508		
Turnover intention	Turnover 1	0.671	0.450	0.727	0.476
	Turnover 2	0.658	0.433		
	Turnover 3	0.751	0.564		

In this study, we employed the Heterotrait-Monotrait Ratio (HTMT) to assess the discriminant validity of the measurement model. HTMT is a correlation-based method used to evaluate the degree to which latent constructs are distinct from one another. According to the recommendations of Henseler et al. (2015), an HTMT value below 0.85 indicates satisfactory discriminant validity, suggesting that different latent constructs can be effectively distinguished. The HTMT values calculated in this study were all below 0.85, as detailed in Table 3. This result demonstrates that the latent variables exhibit good discriminant validity, meaning that the relationships among different constructs are not excessively high. Consequently, the findings support the discriminant validity of the measurement model.

**Table 3:** Heterotrait-monotrait ratio.

**Variable**	**Workload**	**Emotional demands**	**Job burnout**	**Turnover intention**
Workload	-			
Emotional demands	0.290	-		
Job burnout	0.349	0.372	-	
Turnover intention	0.208	0.150	0.396	-

### 4.2 Descriptive statistics and correlation

Table 4 presents the means, standard deviations, and correlation coefficients of all variables in this study. The average workload level was relatively high (M = 5.55, SD = 1.05), indicating that childcare teachers generally perceived their work as demanding. Emotional demands also scored high (M = 6.08, SD = 0.78), suggesting that participants frequently experienced emotional challenges when interacting with children, parents, and colleagues. Job burnout showed a moderate level (M = 5.61, SD = 0.87), reflecting that respondents occasionally felt exhausted or emotionally drained at work. The mean score of turnover intention was also moderate to high (M = 5.89, SD = 0.76), implying that some teachers had considered leaving their current positions.

**Table 4:** Descriptive statistics and correlation.

**Variable**	**M**	**SD**	**(1)**	**(2)**	**(3)**	**(4)**	**(5)**	**(6)**
(1) Age	2.530	1.169	-					
(2) Job tenure	2.620	1.202	0.422^***^	-				
(3) Position	3.420	0.932	0.572^***^	0.473^***^	-			
(4) Workload	5.550	1.047	0.009	0.022	−0.039	-		
(5) Emotional demands	6.082	0.784	−0.042	0.026	−0.051	0.267^***^	-	
(6) Job burnout	5.612	0.874	−0.234^**^	−0.174^*^	−0.164^*^	0.324^***^	0.342^***^	-
(7) Turnover intention	5.890	0.762	−0.072	−0.112	−0.031	0.213^**^	0.133	0.337^***^

^***^p < 0.001.

^**^p < 0.01.

^*^p < 0.05.

In addition, workload was significantly positively correlated with emotional demands (*r* = 0.267, *p* < 0.001), job burnout (*r* = 0.324, *p* < 0.001), and turnover intention (*r* = 0.213, *p* < 0.01). Emotional demands were also significantly positively correlated with job burnout (*r* = 0.342, *p* < 0.001), and job burnout was significantly positively correlated with turnover intention (*r* = 0.337, *p* < 0.001). However, the correlation between emotional demands and turnover intention was not significant (*r* = 0.133, *p* > 0.05). Overall, these findings generally support the hypothesized relationships proposed in this study.

### 4.3 Results of hierarchical regression and hypothesis testing

Prior to hypothesis testing, a model fit analysis was conducted. The results indicated that the model fit indices met the recommended thresholds (χ^2^ = 310.114, SRMR = 0.054, RMSEA = 0.051, CFI = 0.951, TLI = 0.944; IFI = 0.951), demonstrating a good fit with the theoretical model. Following the confirmatory factor analysis (CFA) for the measurement model, the proposed theoretical model and its measurement variables exhibited strong reliability and validity, allowing the study to proceed with hypothesis testing.

Model 2 in Table 5 shows that, after controlling for the relevant variables, both workload (β = 0.257, *p* < 0.001) and emotional demands (β = 0.270, *p* < 0.001) have significant positive relationships with job burnout, thus supporting Hypotheses H1 and H2. In Model 4, after including control variables and the mediating variable, workload (β = 0.196, *p* < 0.01) has a significant positive relationship with turnover intention, while emotional demands (β = 0.007, *p* > 0.05) do not significantly impact turnover intention. However, job burnout exhibits a significant positive relationship with turnover intention (β = 0.290, *p* < 0.001).

**Table 5:** Hierarchical regression.

**Variable**	**Job burnout**		**Turnover intention**			
	**Model1**	**Model2**	**Model3**	**Model4**	**Model5**	**Model6**
Age	−0.0187^*^	−0.186^*^	−0.051	−0.055	−0.001	−0.018
Job tenure	−0.083	−0.114	−0.114	−0.129	−0.096	−0.105
Position	−0.018	0.020	0.052	0.073	0.067	0.071
Workload		0.257^***^		0.196^**^	0.122	−0.077
Emotional demands		0.270^***^		0.085	0.007	0.031
Job burnout					0.290^***^	0.305^***^
Workload x job burnout						0.249^*^
Emotional demands x job burnout						−0.020
*R^2^*	0.061	0.235	0.015	0.069	0.133	0.156
*ΔR^2^*	0.047	0.216	0.000	0.045	0.106	0.121
*F*	4.247	11.949	0.987	2.885	4.950	4.418

^***^p < 0.001.

^**^p < 0.01.

^*^p < 0.05.

According to MacKinnon et al. (2007) mediation testing procedure, mediation is confirmed when the direct effect of the independent variable on the mediating variable is significant, and the direct effect of the mediating variable on the dependent variable remains significant after controlling for the independent variable. In this study, job burnout mediates the relationships between workload and turnover intention, as well as between emotional demands and turnover intention.

### 4.4 Mediation effect

To further verify the significance of the mediating effect of job burnout, Hayes (2013) PROCESS Model 4 was applied with 5,000 bootstrap samples to calculate the confidence intervals for the indirect effects. If the confidence interval does not include zero, the indirect effect is deemed significant, confirming the presence of mediation (Hayes, 2013). The results show that the confidence interval for the indirect effect of job burnout between workload and turnover intention does not include zero [coefficient = 0.069, SE = 0.033, 95%CI (0.023, 0.152)], confirming the mediating effect and supporting Hypothesis H3. Similarly, the confidence interval for the indirect effect of job burnout between emotional demands and turnover intention also does not include zero [coefficient = 0.106, SE = 0.031, 95%CI (0.051, 0.172)], thus supporting Hypothesis H4.

### 4.5 Moderating effect

To test the moderating effect of job burnout and avoid multicollinearity issues, this study followed Aiken and West (1991) recommendation of standardizing the independent and moderating variables by centering the means to zero (Centering to Means) and then calculating the interaction term. If the interaction term is significant, an interaction graph is plotted to examine whether the interaction pattern supports the hypothesis.

As shown in Table 4, after controlling for age, job tenure, and position, the interaction term “workload × job burnout” is significant (β = 0.249, *p* < 0.05), indicating that job burnout moderates the relationship between workload and turnover intention. However, the interaction term “emotional demands × job burnout” is not significant (β = −0.020, *p* > 0.05), meaning that Hypothesis H6 is not supported.

**Figure 2:**
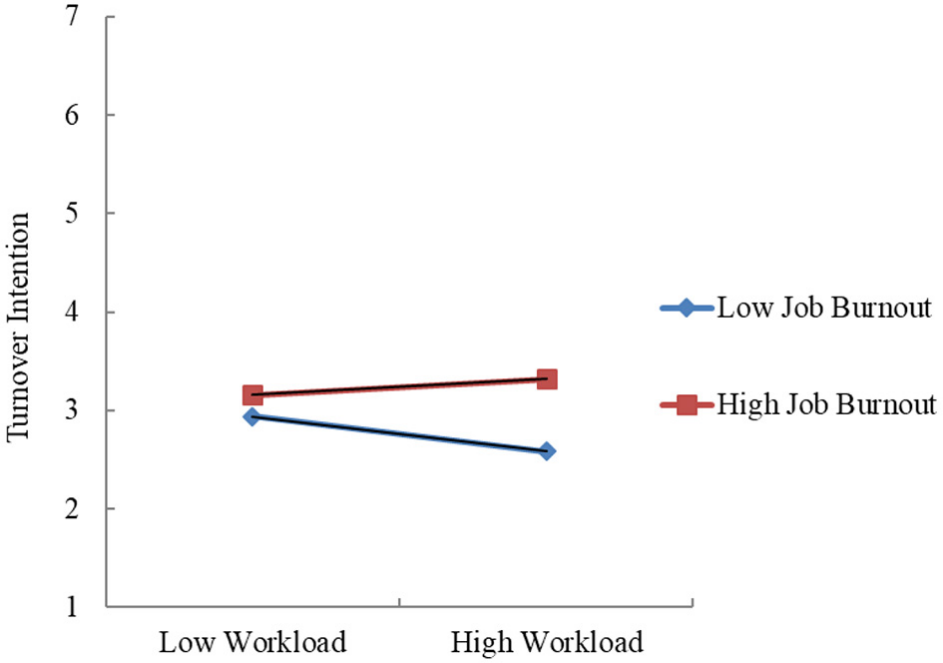
Interaction plot of workload and job burnout.

To further explore the significant interaction, Hayes (2013) PROCESS Model 1 was used with 5,000 bootstrap samples to calculate the confidence interval. The results show that the confidence interval for the interaction effect of “workload × job burnout” does not include zero (coefficient = 0.132, SE = 0.060, 95%CI [0.014, 0.251]), confirming the presence of the moderating effect. Finally, an interaction plot was created to illustrate the moderation effect. As shown in Figure 2, when job burnout is high, the relationship between workload and turnover intention is stronger, while when job burnout is low, this relationship is weaker. This finding supports Hypothesis H5.

Table 6 summarizes the results of the hypothesis testing. As shown, all direct and mediating effects (H1–H4) were supported, indicating that both workload and emotional demands significantly increased job burnout, which in turn heightened turnover intention. These findings are consistent with the theoretical expectations based on the JD-R model, suggesting that excessive job demands deplete employees' psychological resources and consequently lead to withdrawal intentions.

**Table 6:** Summarizes the results of the hypothesis.

**Hypotheses**	**Statement**	**Result**
H1	Workload has a significant positive impact on job burnout.	Supported
H2	Emotional demands have a significant positive impact on job burnout.	Supported
H3	Workload has a significant positive impact on turnover intention through job burnout.	Supported
H4	Emotional demands have a significant positive impact on turnover intention through job burnout.	Supported
H5	Job burnout moderates the relationship between workload and turnover intention.	Supported
H6	Job burnout moderates the relationship between emotional demands and turnover intention.	Unsupported

Regarding the moderating effects, job burnout significantly moderated the relationship between workload and turnover intention (H5), implying that employees with higher levels of burnout are more likely to exhibit turnover intentions when facing heavy workloads. However, the moderating effect of job burnout on the relationship between emotional demands and turnover intention (H6) was not significant. This suggests that while emotional demands contribute to burnout, their direct influence on turnover intention may be less dependent on burnout levels. Overall, the results provide partial support for the proposed moderated mediation framework, confirming the dual role of job burnout as both a mediator and a moderator.

## 5 Discussion

First, the finding that emotional demands had a stronger impact on job burnout than workload underscores the central role of emotional labor in preschool teachers settings. This aligns with previous research by Näring et al. (2006) and Yin et al. (2023), who emphasized the emotionally taxing nature of childcare work, often involving continuous emotion regulation, empathy, and affective display (Brotheridge and Grandey, 2002; Lee, 2019). Importantly, this finding extends the JD-R theory, suggesting that not all demands exert equal pressure: emotional demands may act as “high-strain” demands due to their cumulative depletion of emotional and psychological resources, making them particularly potent predictors of burnout in caregiving professions.

In contrast, although workload showed a weaker effect on burnout compared to emotional demands, it remains a significant predictor, echoing prior studies (Creagh et al., 2023; Heffernan et al., 2022). These results support the core JD-R proposition that job demands when not counterbalanced by adequate resources lead to energy depletion and eventual disengagement (Bakker and Demerouti, 2017, 2024). However, our findings add nuance by showing that emotional demands may trigger more intense burnout responses than quantitative workload, calling for a differentiated view of demand types within the JD-R theory.

Second, consistent with Rajendran et al. (2020) and Lee (2019), our findings confirm that job burnout fully mediates the effects of both workload and emotional demands on turnover intention, reinforcing the central mechanisms proposed by both the JD-R theory and the COR theory. According to JD-R theory (Bakker and Demerouti, 2017), excessive job demands—such as workload and emotional strain—deplete employees' personal and psychological resources, leading to burnout and subsequently increasing withdrawal tendencies. From the perspective of COR theory (Hobfoll, 1989; Hobfoll et al., 2018), this process reflects a downward spiral of resource loss in which continuous depletion of emotional and psychological energy reduces individuals' coping capacity and resilience, ultimately resulting in higher turnover intentions. By simultaneously considering both physical (workload) and emotional (emotional demands) aspects of job demands, this study highlights how resource depletion operates across multiple domains, providing a more comprehensive understanding of burnout's mediating role within the JD-R and COR theoretical frameworks.

Third, this study identifies job burnout as not only a mediator but also a moderator, particularly in the relationship between workload and turnover intention. Consistent with Chen et al. (2025), who emphasized the moderating role of burnout in the stressor–strain process, our results further demonstrate that burnout amplifies the impact of job demands on turnover intention. Specifically, when burnout levels are high, the effect of workload on turnover intention becomes more pronounced, aligning with the “resource loss spiral” proposed by COR theory (Hobfoll et al., 2018). This finding suggests that burnout produces a compounding effect: as personal resources are depleted, individuals become increasingly susceptible to additional stressors, accelerating the deterioration of well-being and work-related outcomes. The dual role of burnout—both as a mediator and a moderator—has been largely underexplored in prior research. Thus, this study contributes novel insights by showing that burnout is not merely a byproduct of excessive demands but also an amplifying mechanism that intensifies their negative consequences, particularly under prolonged exposure to high workloads.

Interestingly, the moderating effect of burnout was not observed in the relationship between emotional demands and turnover intention. This divergence from expectations suggests that emotional demands may interact with more complex personal and contextual factors, such as emotion regulation strategies, individual resilience, or social support. It also raises the possibility that some individuals may develop adaptive coping mechanisms for emotional labor, as suggested by studies on emotional intelligence and resilience in caregiving professions (Brotheridge and Lee, 2002; Grandey, 2000). Future research may benefit from incorporating such personal resource variables into the model to better capture differential responses to emotional demands.

## 6 Conclusion

This study examined how job demands, specifically workload and emotional demands, influence turnover intention among preschool teachers, with job burnout serving as both a mediator and a moderator. Grounded in the JD-R theory, the findings revealed that both types of demands affect turnover intention only through burnout. This supports the idea that burnout fully mediates the link between excessive demands and adverse organizational outcomes. The results highlight that job demands must first manifest as psychological strain, such as emotional exhaustion or depersonalization, before leading to turnover intention.

In line with the COR theory, the moderation analysis showed that burnout intensifies the relationship between workload and turnover intention. This is consistent with the concept of a resource loss spiral. When burnout is high, the impact of workload on turnover intention is amplified. This suggests that burnout reflects cumulative resource depletion and reduces individuals' coping capacity. However, no moderating effect was found between emotional demands and turnover intention. This finding suggests that emotional labor may involve more complex or individualized mechanisms beyond the current model.

These findings underscore the dual role of burnout in the stress–turnover process. From a practical perspective, they highlight the need for early interventions focused on managing workload and providing emotional support to prevent burnout and reduce turnover among preschool teachers.

### 6.1 Theoretical implications

This study offers several theoretical contributions. First, by identifying job burnout as both a mediator and a moderator, it extends prior applications of the JD-R and COR theories. While most JD-R-based studies have focused on a linear pathway from job demands to burnout to outcomes, this study emphasizes the reciprocal and amplifying role of burnout. According to COR theory's resource loss spiral, burnout is not only a result of job demands but also a factor that increases vulnerability to further stressors.

Second, the findings highlight the differential effects of demand types and support a more nuanced application of the JD-R theory. Emotional demands, due to their invisible, ongoing, and relational nature, appear to cause greater emotional strain than workload. This suggests that future models should distinguish among emotional, cognitive, and physical demands instead of treating all demands as a single construct.

Third, the study proposes that interventions should not only focus on increasing job resources, as commonly emphasized in JD-R theory literature, but also address the need to interrupt the burnout spiral. This is especially important for preschool teachers already experiencing high levels of exhaustion. This reconceptualization of burnout positions it as a dynamic condition that shapes how job demands are perceived and how they influence behavioral outcomes, such as turnover intention.

### 6.2 Managerial implications

From a practical standpoint, school administrators and managers must recognize the influence of both workload and emotional demands on preschool teachers' burnout and turnover intention. Proactive strategies are needed to address these risks.

First, high workload not only increases stress directly but also leads to turnover intention through job burnout. Managers should reduce unnecessary tasks, distribute workloads more efficiently, and make adjustments to help alleviate burnout and reduce turnover.

Second, even though burnout did not moderate the link between emotional demands and turnover intention, emotional labor remains a significant source of stress. Providing emotional support systems, offering emotion regulation training, and encouraging healthy emotional expression may help reduce the long-term burden of emotional labor.

Finally, this study highlights the dual role of burnout. This reinforces the need for early detection and preventive systems. Schools should implement mechanisms to monitor burnout, offer timely psychological support, and allocate appropriate resources to minimize the risk of escalation. These strategies can improve teacher well-being, reduce turnover, and create a healthier work environment.

### 6.3 Research limitations and future research developments

Despite its contributions, this study has several limitations. First, all data were collected via self-reported questionnaires, which may introduce common method variance (CMV). Although procedural remedies were applied, such as ensuring anonymity, item separation, and temporal spacing (Podsakoff et al., 2003), and Harman's single-factor test indicated minimal CMV concerns, bias cannot be entirely ruled out. Future studies should employ multi-wave or longitudinal designs to examine the temporal dynamics of burnout and turnover, thereby strengthening causal inference. Additionally, adopting advanced statistical approaches such as the marker variable technique (Podsakoff et al., 2024) could further enhance CMV control and improve result robustness.

Second, while the variables selected were theoretically grounded, they may not fully capture the complexity of preschool teachers' turnover intentions. This study primarily focused on workload, emotional demands, and job burnout. However, other factors—such as role overload, career plateau, effort–reward imbalance, role ambiguity, and work–family conflict—may also influence turnover intention. Future research should incorporate broader job demands, personal and organizational resources (e.g., psychological capital, social support, leadership), to develop a more comprehensive model.

Third, the study employed a convenience sampling method, which may limit the generalizability of the findings. Participants were drawn from a specific professional network of preschool teachers in Taiwan. Future studies could collect data from different regions and educational settings to increase sample diversity and enhance the external validity of results.

## Data Availability

The raw data supporting the conclusions of this article will be made available by the authors, without undue reservation.
